# Detecting and Comparing Non-Coding RNAs in the High-Throughput Era

**DOI:** 10.3390/ijms140815423

**Published:** 2013-07-24

**Authors:** Giovanni Bussotti, Cedric Notredame, Anton J. Enright

**Affiliations:** 1 European Bioinformatics Institute, Wellcome Trust Genome Campus, Hinxton, Cambridge CB10 1SD, UK; E-Mail: aje@ebi.ac.uk; 2 Bioinformatics and Genomics Program, Centre for Genomic Regulation (CRG), Aiguader, 88, 08003 Barcelona, Spain; E-Mail: Cedric.Notredame@crg.eu

**Keywords:** lncRNAs, ncRNAs, high-throughput, RNA-seq, comparative biology, sequencing

## Abstract

In recent years there has been a growing interest in the field of non-coding RNA. This surge is a direct consequence of the discovery of a huge number of new non-coding genes and of the finding that many of these transcripts are involved in key cellular functions. In this context, accurately detecting and comparing RNA sequences has become important. Aligning nucleotide sequences is a key requisite when searching for homologous genes. Accurate alignments reveal evolutionary relationships, conserved regions and more generally any biologically relevant pattern. Comparing RNA molecules is, however, a challenging task. The nucleotide alphabet is simpler and therefore less informative than that of amino-acids. Moreover for many non-coding RNAs, evolution is likely to be mostly constrained at the structural level and not at the sequence level. This results in very poor sequence conservation impeding comparison of these molecules. These difficulties define a context where new methods are urgently needed in order to exploit experimental results to their full potential. This review focuses on the comparative genomics of non-coding RNAs in the context of new sequencing technologies and especially dealing with two extremely important and timely research aspects: the development of new methods to align RNAs and the analysis of high-throughput data.

## 1. Introduction

### 1.1. The Non-Coding RNA (New)-World

**Figure 1:**
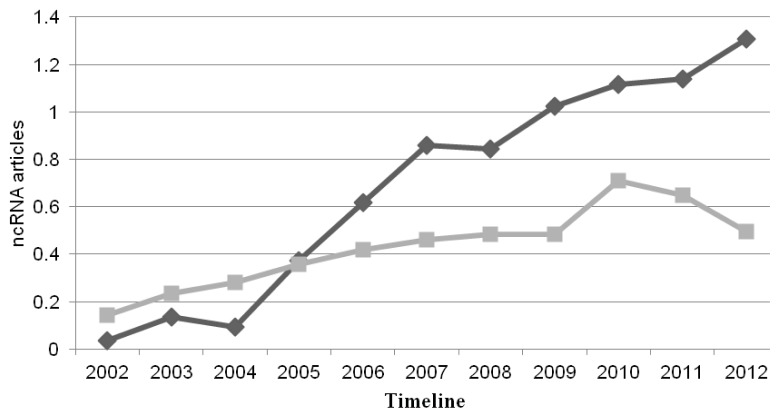
Number of publications in PubMed found using the keyword “ncRNA” (dark grey) and “regulatory RNA” (pale gray). The x-axis represents the timeline, the y-axis the number of times the words “ncRNA” and “regulatory RNA” match a publication in PubMed normalized by the total number of publications in that year (expressed as one part per ten thousand).

In recent years, the non-coding RNA (ncRNA) field has rapidly expanded (Figure 1) with a rapid increase in the number of newly identified and biologically relevant ncRNAs. Just a decade ago, the number of known ncRNAs was restricted to a small amount of housekeeping genes (including ribosomal RNAs, transfer RNAs and small nucleolar RNAs) and an even more limited collection of regulatory RNAs, such as lin-4 in *Caenorhabditis elegans* [1] and Xist in mammals [2]. Since then, the number of novel ncRNAs has increased dramatically and far more is known about their function, biogenesis, length, structural and sequence features. New and ever more sophisticated high-throughput technologies, such as tiling arrays and next generation sequencing (NGS) have been applied to comprehensively profile the transcriptome of various organisms.

This wealth of data has allowed the identification of thousands of novel short ncRNAs, including PIWI interacting RNAs [3] and small nucleolar RNAs [4] and has resulted in the compilation or the update of many publicly available databases [5–10]. Furthermore, high-throughput approaches have revealed extensive and pervasive transcription of long ncRNAs (lncRNAs) [11–13], operationally defined as functional RNA longer than 200 base pairs that does not template protein synthesis. In the human genome, for instance, the GENCODE consortium annotated 9,640 lncRNA loci representing 15,512 transcripts [3,14] and in [15] the authors estimated that total number of human lncRNAs genes to be about 50,000, more than two-fold greater than the number of protein-coding genes. These discoveries were very timely in the context of growing concern for the lack of a significant correlation between the number of protein-coding genes and the commonly accepted concept of “organism complexity” [4,16,17]. It was proposed that alternative splicing and ncRNAs could be accountable for complex gene regulation architectures, meaning that the “Central Dogma” of genetic programming enunciated by Francis Crick in 1958 (RNA is transcribed from DNA and translated into protein) [18] had to be slightly altered and at least in higher eukaryotes may be inadequate [16,17]. The biological role of most of these novel long untranslated molecules is still a controversial issue. Some authors have even raised doubts about whether these transcripts are functional at all [19]. The lack of shared discernible features hampers our ability to define lncRNA classes, thus impeding function prediction [20]. However mounting experimental evidence illustrates that lncRNAs are implicated in a variety of biological processes [21] and are linked to various diseases including cancer [22]. Additionally, the functional roles of lncRNA transcripts have been uncovered in signaling sensors [23], embryonic stem cell differentiation [11], brain function [24,25], subcellular compartmentalization and chromatin remodeling [26]. Some examples include X chromosome inactivation by Xist, the silencing of autosomal imprinted genes accomplished by Air, nuclear trafficking regulated by NRON and muscle differentiation controlled by linc-MD1 [2,27–29]. In [30] the authors identified a class of lncRNAs named ncRNA-a (ncRNA-activator) able to stimulate the expression of proximal protein-coding genes, and a recent update on ncRNA-a [31] showed that the co-activator complex Mediator plays a central role in the activation process. See [21] and [32] for more examples and lncRNAdb [33] for the central repository of known lncRNAs in eukaryotes. lncRNAs are expressed, some are spliced, they are often conserved across vertebrates, and their expression is frequently tissue- and/or cell-specific and localized to specific subcellular compartments [11,25,34]. It has been shown that lncRNAs can act both in *cis* [30,35] and in *trans* [36], some acting as precursors for short ncRNAs [37–39], while others act independently as long transcripts. As in [40] lncRNAs can be classified as “intergenic” or “genic” depending on their position/orientation with respect to protein-coding genes. lncRNAs not overlapping any protein-coding gene are tagged as intergenic and then further classified according to their transcription orientation with the closest protein-coding loci (same sense, convergent, or divergent). The genic lncRNA set are catalogued as “exonic” if overlapping a protein-coding exon. Otherwise, lncRNAs are labeled as “intronic”, when positioned within protein-coding introns or as “overlapping”, in presence of a protein-coding transcript located within the intron of the lncRNA [40].

### 1.2. lncRNA Challenges

Although the conservation level of different lncRNAs may be not always directly comparable (e.g., the evolutionary conservation of genic lncRNAs may be biased by the presence of the protein-coding genes), overall approximately half of reported human lncRNA exhibit significant conservation across mammals [40]. These levels suggest some key cellular function, even though only a small fraction of these transcripts have so far been functionally characterized. Such functional analyses remain however, very superficial and lack precise molecular mechanisms explaining the activity of these novel transcripts. Our low level of understanding can be in part attributed to the difficulty with working experimentally with lncRNAs: detection is difficult for a combination of biological and technical aspects. The first relates to the low levels of non-coding genetic expression. After ribosomal RNA (rRNA), protein-coding mRNA represents the highest population of RNA species [41]. In previous studies [34,42,43] it has been reported that lncRNAs are on average 3 to 10 fold less expressed than mRNAs. Besides the complicated task of capturing weaker expression signals, many lncRNAs have pronounced tissue/stage specificity [43,44]. In other words, lncRNA genes can easily be left undetected unless the correct cell type and condition are considered. One more complication for ncRNA discovery has been the difficulty of sequencing deep enough, a hurdle only recently overcome by NGS. Additionally, our ability to assemble and annotate genomes was less advanced than currently and we had simplified notions of transcriptome complexity. Most of the classical low-throughput approaches, such as RT-PCR and northern blotting, have been successfully used to analyze the expression of small numbers of genes, but they were not adequate to address the “pervasive transcription” aspect of genomes [45,46]. Furthermore, there are specific classes of ncRNAs, such as circular RNAs (circRNAs), that have been extremely hard to identify. circRNAs are a class of non-coding RNA family that were discovered more than 20 years ago [47–50]. These RNAs form circles that arise from non-canonical splicing events (also known as exon shuffling) that join a splice donor to an upstream splice acceptor to produce a circular RNA molecule. Recent studies [51,52] show that the human circRNA CDR1as, antisense to the Cerebellar Degeneration-Related protein 1 (CDR1), hosts around 70 binding sites for the miR-7 microRNA and is highly associated with the Argonaute protein Ago2 as demonstrated by PAR-CLIP and HITS-CLIP experiments [51,52]. Mainly because of their non-canonical splicing behavior, circRNAs have eluded detection by next generation sequencing until recently. These latest studies adopted a novel computational approach to identify circRNAs from high-throughput RNA-seq data and demonstrated their widespread abundance within transcriptomes [51,53].

**Figure 2:**
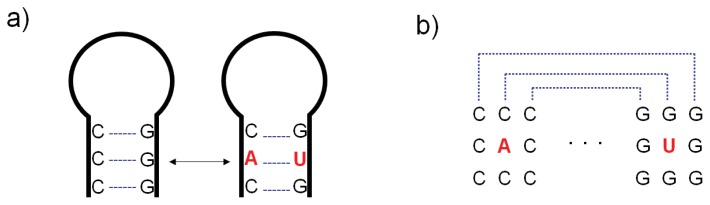
RNA mutations are tightly linked to the RNA structure conservation. (**a**) Example where the mutation of a C into an A is compensated by the change G–U. The two positions are not independent, but communicating one with the other to maintain the structure unvaried; (**b**) Same hairpin as shown in (a). The presence of the compensatory mutation is highlighted by the multiple sequence comparison.

In general, a major obstacle for ncRNA detection is the difficulty to perform informative sequence comparisons. Standard primary sequence alignment is hampered by the low complexity of the nucleic alphabet, making it difficult to produce statistically meaningful RNA alignments. Ribonucleic acid chemistry relies on just four primary residues: two purines and two pyrimidines. Consequently, RNA gene sequences do not have strong statistical signals, unlike protein-coding genes. For instance two RNA sequences must share an identity of at least ~60% to be considered significant in homology relationships prediction [54]. Below this level, common ancestry is hard to infer with certainty. By comparison, this threshold is around ~20%–35% for proteins [55]. Furthermore, ncRNA appears to be evolving rapidly [56] or are under the influence of very specific evolutionary constraints [56]. It was proposed that most ncRNAs evolve at higher mutation rates, with the maintenance of secondary structures being the main source of selection [57,58]. This assumption makes sense from an evolutionary standpoint. As ncRNAs will be left untranslated, the nucleotide sequence itself is not constrained to keep the codon reading frame. Of course many exceptions exist. Specific ncRNAs types can hold functional sequences and act via their primary sequence (*i.e*., miRNAs). Previous reports have shown that at least some miRNA genes are well conserved across species [59–61], reinforcing the idea that sequences encoding a function evolve under purifying selection. Aside from these specific and relatively rare examples, it seems that for most known ncRNAs, evolution is limited by structural constraints [62,63]. This induces a characteristic pattern of covariance that occurs when a mutation is affecting a nucleotide pairing to another in a structured domain (Figure 2). If the mutation breaks the base pairing so that the functionality of such a domain is compromised, the matching nucleotide is favored to mutate in turn, *i.e.*, is co-varying to restore the base pairing and keep the structure unchanged.

For most aligners these features of RNA are hard to account for when using standard alignment procedures that postulate positional independence and seek only to maximize identity. Furthermore, RNA can hold functional pseudo-knots. These are structural configurations where at least two RNA stem-loops are interposed one into the other. Although some comparative approaches including pseudo-knots exist [64,65], these are disregarded by most software due to reasons of computational complexity [66]. As a consequence ncRNA sequences are harder to align than proteins, a limitation that affects our ability to accurately detect and classify them. The difficulty in comparing ncRNAs calls for other information sources that alignment algorithms can use. More than ever, the issue of accurately comparing and aligning ncRNAs is of critical importance. This is precisely the problem discussed in the following section, where we review established and more recent methodologies able to make the best of available RNA information (Section 2). Next we discuss different homology based strategies for ncRNA detection (Section 3) and the analysis of high-throughput expression data (Section 4). See Table 1 for a summary of the resources described in the text.

## 2. Comparing Non-Coding RNAs

As mentioned, generating meaningful ncRNA alignments is a challenging task and at least in some cases, the best accuracy could be achieved by exploiting RNA structural information. However, in many situations using such information is complicated. In spite of the development of aligners that take into account the RNA secondary structure information, one major issue is the poor availability of high quality structures. The problem is at least in part due to the difficulties encountered at experimental level in crystallization. Getting crystals from RNA molecules is complicated because of their chemical specificity. The accumulation of crystals is prevented by the high RNA flexibility. RNAs have flexible structures adopting inter-domain movements and with respect to proteins have weaker tertiary interactions [67]. The polyanionic charge of the phosphate backbone makes the nucleotide sequence move much more than in proteins and this makes the packaging of crystals much harder to achieve. As a consequence, the crystals are either hard to grow or uninformative. Even when trying to resolve RNA molecules in solution using NMR, the resonance assignment is more difficult for RNA than for proteins [68]. RNAs have only 4 primary nucleosides instead of the 20 different amino-acid side chains found in proteins [69]. Thus, the chemical shift dispersion is narrower in RNA than in proteins, resulting in less informative spectra [69].

### 2.1. RNA Structure Prediction

Because of these limitations, RNA structure is usually computationally predicted without any experimental support [70,71]. RNA secondary structure inference amounts to the computation of base-pairings that shape the *in vivo* molecule structure. The prediction is performed using primary RNA sequence data. Another possibility is including other sources of statistical information to constrain structure prediction, for instance an alignment of structurally homologous RNA sequences. Regarding single sequence RNA secondary structure predictions, there are two main groups of approaches: empirical free-energy parameters [72] and knowledge based [73–75]. The first considers a biophysical model to calculate the structure whose folding has the minimum Gibbs free energy (ΔG). In this approach, [76–80] the nearest stable folding is employed to compute the conformational stability of the Minimum Free Energy (MFE) structure. The energy parameters needed in this approach were assessed on a set of optical melting experiments on model systems [77–79]. The two most popular implementations of the MFE structure prediction algorithm are Mfold [70] and RNAfold [81] packages. The latter implements McCaskill’s algorithm [82], an approach to calculate the probability of a certain secondary structure in the whole thermodynamic ensemble. This approach is based on the partition function, which sums all Boltzmann weighted free energies of each secondary structure that is possible given an RNA sequence. In this model, the confidence estimate in a particular base pair *i*,*j* is given by the sum of the probabilities of all structures containing that base pair *i*,*j* divided by the sum over all structures [83]. Knowledge based approaches rely on probabilistic models, where statistical learning procedures are used instead of empirical measurement of thermodynamic parameters. The Stochastic Context Free Grammar (SCFG) model [73] represents one popular example of such probabilistic models. The parameters used by the SCFG models are estimated on the set of RNAs with known structures (e.g., rRNA).

**Figure 3:**
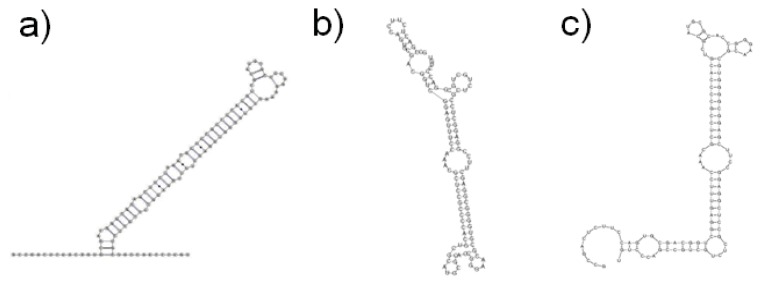
Consistency of RNA secondary structure predictions. In this example the human mir-3180 (Rfam accession id RF02010; AJ323057.1/363-249) was folded using different approaches yielding different output structures. (**a**) Secondary structure of the family as estimated by Rfam release 10.1; (**b**) RNAfold web server prediction based on Vienna RNA package version 2.0.0. [93]; (**c**) Mfold web server prediction, running Mfold version 4.6 [71].

Prediction consistency is the main limit of both MFE and knowledge based methods [84]. (See the example in Figure 3). The percentage of known base pairs predicted correctly by the secondary structure prediction methods ranges from 40% to 75% [73–75,85]. This low figure may be the result of three confounding factors. Firstly, folding *in vivo* can be influenced by RNA chaperones [86], RNA editing [87], and even by the transcriptional process itself [88]. At present, there is no software able to account for these effects. Secondly, looking for a single structure can sometimes be inadequate. There are cases, such as the ribo-switches [89,90], where multiple functional structures can be derived from the same sequence depending on conditions such as temperature or other external factors. Standard predictors are not well suited to deal with such situations and require dedicated tools able to identify potential conformational switches [91,92]. Thirdly, RNAs might contain pseudo-knots, which are ignored by most tools due to reasons of computational complexity [66].

The best secondary structure prediction accuracy can be achieved using comparative methods [66]. These apply to a set of structurally homologous RNA sequences being aligned. For some of these computation tools, the output will be the prediction of each individual homologous structure, while in other situations the result will be a unique consensus structure. The consensus structure does not exist *in vivo*, but rather it is a model that captures the common, relevant structural aspects conserved within the family.

### 2.2. Structure Prediction and Alignment Strategies

Due to the close relationship between sequence and structure, structure prediction and sequence alignment can be described as interdependent problems [63]. As theorized by Sankoff [94], the most suitable approach should involve the simultaneous alignment and folding of the considered sequences. Unfortunately, a strict application of this approach would be computationally prohibitive and the lack of an appropriate heuristic solution is reflected by the wealth of available alternative solutions. The web server WAR [95] is a good example. This tool allows the execution of a total of 14 different strategies to align and predict the common secondary structure of multiple RNA sequences. Over the years, so many methods have been described that some kind of classification is needed to catalogue them. Paul Gardner proposed three categories he refers to as “plans” [66,96]. In plan A, one starts with the estimation of a multiple sequence alignment and then the aligned sequences are folded jointly (as a kind of consensus). The initial alignment can be done by any standard MSA aligner (e.g., ClustalW [97], T-Coffee [98]), and the folding of the aligned sequences can be performed by a plethora of tools (e.g., RNAalifold [99], PFOLD [100], ILM [101], ConStruct [102]) optimizing a score based on compensated mutations and thermodynamics. However this strategy is effective just in a determined sequence similarity range. On one hand, sequences that are too similar do not carry any covariance or purifying selection information and are less informative from an evolutionary standpoint. On the other hand, sequences need to be similar enough to be meaningfully aligned as below the “twilight zone” of similarity sequence alignment tends to obscure the covariance signal [96]. Plan B makes it possible to use evolutionary signals to help improve the reliability of structure predictions. This approach accounts for an underlying RNA substitution model where mutation probabilities are affected by structural dependencies. The maintenance of a 3D fold is a major evolutionary constraint influencing the acceptance of point mutations. From this perspective, a nucleotide located in the stem is not as free to mutate as a nucleotide located in a loop. Substitutions taking place in structured functional domains of RNAs can disrupt the wild-type conformation and seriously affect the molecular function. As a consequence, a nucleotide whose pairing has been disrupted by the mutation of its mate, is more likely to mutate itself so as to recover the original structure and rescue the function. Back in 1985 Sankoff developed a dynamic programming algorithm able to take into account sequence and structure of an RNA molecule simultaneously [94]. Unfortunately this algorithm is computationally expensive, with a running time equal to O(*N*^3^*^m^*), where *m* is the number of sequences and *N* their length. This means that a pairwise comparison has the tremendous CPU cost of O(*N*^6^) which makes this algorithm inapplicable most of the times and calls for simplified heuristics. Several banded modifications of the Sankoff algorithm impose limits on the size or shape of substructures, like Dynalign [103,104], Foldalign [105,106], Stemloc [107], Consan [108]. Another example is pmmulti [109], a Sankoff algorithm variant able to perform multiple secondary structure alignments by aligning consensus base pair probability matrices. Plan C is used by programs such as R-Coffee [110] or RNAcast [111]. In these methods each individual sequence is folded separately before running the alignment. Equivalent secondary structures between two RNAs can be used to enhance the alignment accuracy. For instance, let seq1 and seq2 be two RNA sequences, *x* and *y* be two nucleotides matching in a secondary structure in seq1, and *x*′ and *y*′ be two nucleotides matching in a secondary structure in seq2. If *x* aligns to *x*′ then implicitly *y* should be driven to align to *y*′. For example, R-Coffee uses RNAplfold [112] to compute the secondary structure of the provided sequences. After that, R-Coffee computes the multiple sequence alignment having the best agreement between sequences and structures. This pre-folding approach is especially valuable when RNAs are too different to be meaningfully aligned merely by using an off-the-shelf multiple alignment tools (*i.e.*, ClustalW [97]). Plan C is particularly well suited to situations where experimental secondary structures are available.

The situation is radically different when experimental 3D structure information is available. In this case the RNA alignment problem becomes similar to the protein structural alignment problem. This problem is nondeterministic polynomial-time complete (NP-complete) and it involves the alignment of two distance matrices. In most cases the problem can be solved in a rather satisfying way by using heuristics making the best of the geometric information contained in the PDB models. Examples of pairwise structural alignment methods for RNA are SARA [113], DIAL [114], iPARTS [115], ARTS [116] and SARSA [117]. Besides this, recently several 3D RNA structure database search programs were developed, such as LaJolla [118] and FRASS [119].

Giving an exhaustive overview of the methods available for folding and aligning structured RNA sequences is well beyond the scope of this review. Over the last twenty years, more than 30 methods have been described that deal with these issues which is an indication of the complexity of this problem, despite 25 years of research following its formal description by Sankoff.

## 3. Detecting ncRNA Homologues

In the ncRNA field another critical step is the collection of homologues to genes of interest. Homologues can be used in several situations, such as the detection of functional motifs, inference of possible evolutionary steps or the design of laboratory experiments. For instance, the conservation across species of a certain ncRNA can be used to estimate how likely a gene is to be functionally important. Such information can be used to prioritize experiments, e.g., knockdown experiments of the orthologous gene in a model organism. Over the last few years many different methods have been developed to approach the problem of RNA homology detection. As previously shown [120], homology search methods can be grouped in three sets: sequence-based, profiles and structure-based methods. The first and most straightforward approach to look for homologues is by comparing the nucleotide sequences. Already in 1981 Smith and Waterman developed a dynamic programming algorithm that allows for pairwise local alignment [121]. Nevertheless, this approach is CPU time demanding and implementations of this method have been until recently unpractical for large-scale database and genome wide screenings [122]. For this reason, alternative approaches such as FASTA [123] or BLAST [124] have been frequently preferred. These methods apply heuristics that boost computational speed at the cost of reduced accuracy. In both BLAST and FASTA, the underlying idea is to skip the time consuming comparison of entire query and target sequences, but rather to start identifying short conserved words in a first step called seeding. After that, more accurate time-consuming local alignments are performed. The second class of approaches are based on profiles, including HMMs. Profile HMMs are probabilistic models that are generated out of an input multiple sequence alignment where each position of the alignment is used to estimate nucleotide frequency. These models can be used to screen databases and look for homologs. Profile HMMs are heuristics having usually superior accuracy over methods based on single sequences [125,126]. However, such models have a linear architecture best suitable for modeling linear protein sequences (as opposed to secondary structure relationships). A more appropriate modeling of an RNA alignment should also consider RNA base pair interactions. The best sensitivity can be attained when applying approaches taking into account at the same time sequence similarity and secondary structures, as the Sankoff algorithm does. Unfortunately, the Sankoff algorithm is too computationally demanding, hence the need for approximate heuristic implementations of this exact algorithm. Such approximations include banded Sankoff tools [104,106,108,127], genetic algorithm implementations such as RAGA [128] and covariance models (CMs). CMs are the most commonly used method to carry out efficient database screening and can be described as special form of stochastic context free grammar (profile SCFGs). CMs were first introduced by Sean Eddy in [129] and implemented in Infernal [130]. This and other related applications such as RSEARCH [131] belong to a class of broadly used homology search tools based on the automatic learning of statistical models (the CMs) estimated from an input multiple RNA alignment decorated with the consensus secondary structure. CMs are probabilistic models that can be derived unambiguously out of a structure-annotated sequence alignment and can be used in turn to query a target sequence database to find homologs. Conceptually CMs are similar to profile HMM but able to include RNA base-pairs interactions information. The modeling of such information results in a higher complexity and CMs are represented by a tree-like model architecture, where the tree shape directly mirrors the consensus RNA structure. Unlike HMM states that only allow the emission of matches and indels, CMs embed new states to handle paired/not-paired and directionality information. For instance, in the paired sites, deletions can involve either a single 5′ or 3′ nucleotide, or the complete base pair. The direction also matters for the insertions that can now concern either the 5′ or 3′ ends of a base pair. In order to permit multi-loops, the bifurcation states are incorporated as well. In spite of their superior accuracy, CM cannot be used in all situations and are restricted to the identification of unsplit genes. The mapping of queries composed by multiple exons is impossible due to the impossibility of aligning secondary structures to a target interrupted by introns whose position is unknown. Moreover CMs need to “learn” from a set of homologous transcripts, but the set of sequences required to train the model are not always available. There is some circularity in this problem where the CM is used to search homologs that are themselves needed to estimate the model. Another layer of complexity results from the need to assemble a multiple sequence alignment of homologous sequences, needed to train the CM. In the CM the alignment will be used for a probabilistic description of matches, mismatches, insertions and deletions. However, generating accurate RNA alignments is difficult. In Rfam [132] CMs parameters are trained on high quality alignments (seed alignment) obtained from literature with manual curation. This input is used to estimate CMs, which are then passed to Infernal for homology searches. This CM/Infernal strategy is analogous to HMM/HMMER used for Pfam [133]. An option for spotting promising sequence segments and accelerate the detection procedure is to include a pre-filtering step as done for the Rfam setup [134]. This can be accomplished by means of *ad hoc* algorithms [135], profile HMMs like ML-heuristic [126] or BLAST with relaxed expectation values (*E*-values) to avoid losing sensitivity as achieved in Rfam [136]. A number of studies have been dedicated to the optimization of BLASTn parameters for seeking RNA homologs. For instance, in one study [120,137] the effectiveness of BLAST and other popular homology search methods tuned for ncRNA screenings were benchmarked. In [138], BlastR is introduced, a method that both takes advantage of di-nucleotide conservation and BLASTp as search engine to discover distantly related homologs. BlastR can be mounted on the top of computationally demanding algorithms to serve as a pre-filtering tool. One merit of this approach is that it neither require profiles nor secondary structure information, but relies solely on information encoded in primary nucleotide sequences.

Together with sequence-based, profiles and structure-based methods, another possibility for detecting inter-species homologs involves the use of multiple genome alignments [43]. Once established reciprocity between blocks of genomes belonging to different organisms (*i.e.*, syntenic regions), coordinate transfer from one gene to its homolog is straightforward and implies the projection of corresponding positions. This has been made possible thanks to the availability of genome sequences [139–142] and the development of alignment tools able to detect orthologous genomic regions, *i.e.*, loci that proceeded from the same genomic position in the ancestral genome [143]. Although comparing ncRNAs is currently still a complicated task, there exist several bioinformatics options to workaround the poor sequence conservation and effectively perform homology based prediction of novel ncRNAs.

## 4. High-Throughput Technologies and Genome-Wide Annotation of ncRNAs

### 4.1. Approaches for the High-Throughput Expression Detection

Recent technological advances have allowed the collection of an unprecedented amount of RNA sequence data coming from a wide range of organisms and conditions. For many years the main strategy for transcript discovery had been the sequencing of cloned complementary DNA (cDNA) of expressed sequence tags (ESTs) [144–146]. EST sequencing was then successfully used for the generation of large-scale expression datasets [147], and already by 1991 this approach had been utilized for human gene discovery [148]. Although it is widely acknowledged that ESTs represent a valuable resource to detect gene expression, they also came with severe limitations such as cost and sequencing requirements. Their dependence on bacterial cloning is an important source of bias and contamination that can lead to redundancy and under-representation or over-representation of host-selected transcripts [149–151]. More recently, oligonucleotide microarray technologies have made high throughput expression analysis much more practical, while the even more recently developed RNA-seq technologies promising transcriptomic analysis of unprecedented accuracy thanks to the application of NGS methods to transcriptome sequencing. Microarrays rely on a collection of nucleotide probe spots attached to a solid support. RNAs are labeled with fluorescent dyes, hybridized to the arrays, washed, and scanned with a laser [152]. Such arrays have been used for the investigation of known or predicted genes and have been until recently one of the most widespread technology for transcriptome exploration. Standard expression arrays are affected by several limitations including the hybridization and cross-hybridization artefacts [153–155], dye-based identification problems [156–160] and physical manufacturing restrictions, impeding the detection of splicing events and the discovery of unannotated genes [151]. A variant of traditional expression array is represented by tiling arrays. These are chips that use extremely densely spotted and probes representing overlapping contiguous regions of genome. Several works relying on this technology and aiming at transcript discovery have been published [38,161–164]. However tiling arrays require a substantial quantity of RNA and have further limitations affecting their sensitivity, specificity and the detection of splicing [151]. For instance, as shown in [165], microarrays lack sensitivity for genes expressed either at low or very high levels and if compared with RNA-seq have much smaller dynamic range. As a consequence, microarrays are inadequate for the quantification of both the prevailing RNA classes, and the less abundant ones. For genes with medium levels of expression, RNA-seq and microarrays return comparable results [165–167]. Still, each approach presents very specific advantages and disadvantages. A thorough comparison of these two approaches lies outside the purpose of this text (for reference, see [152,166,168]). Additional methods for high-throughput RNA discovery include the serial analysis of gene expression (SAGE) [169,170], several updated variants such as LongSAGE [171], RL-SAGE [172], SuperSAGE [173] and analogous approaches like the massively parallel signature sequencing (MPSS) [174]. In general, SAGE-like methods consist in the cloning and then the sequencing of short tags (17–25 nucleotides) coming from RNA extract. The resulting tag sequences can be compared against the source genome or a reference RNA database to attain the digital count of transcript quantities. Two other protocols that can be used in combination with high-throughput sequencing are the paired-end ditags (PETs) [175] and the rapid amplification of cDNA ends (RACE) [176–178]. Both approaches can be used to demarcate transcript boundaries, *i.e.*, define start and end of a transcript. Such information is extremely valuable *in situ* ations where the first and last exons can be respectively 5′ and 3′ associated with other transcript isoforms, thus making it difficult to define gene boundaries. Similarly, the cap analysis of gene expression (CAGE) [179,180] is a technique that allows high-throughout profiling of transcriptional starts points. Another promising application for ncRNA discovery, named RNA CaptureSeq, has been recently reported [181]. This approach is able to reach unprecedented sequencing depth. RNA CaptureSeq is inspired from exome sequencing techniques and relies on the use of tiling arrays in order to enrich the population of RNAs one wants to sequence. This enrichment step allows a sequencing depth that would be impossible when dealing with the full transcriptome. Although RNA CaptureSeq is not suited to generate full transcriptome profile, it can be used to target specific genomic sites and detect transcript isoforms expressed at very low abundance. As shown in [181] RNA CaptureSeq can be used to fuel the detection of ncRNAs that are missed by genome-wide standard RNA sequencing.

### 4.2. Datasets

**Figure 4:**
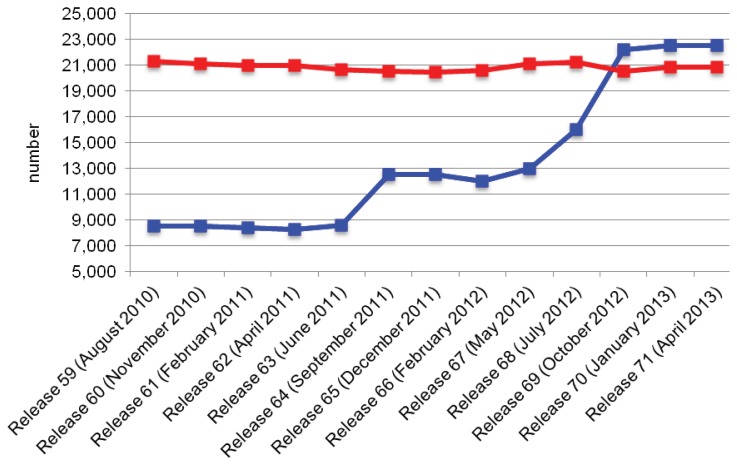
Number of non-coding and protein-coding genes annotated over the last Ensembl releases. The *x*-axis indicates the number and the date of the release. The vertical axis reports the number of ncRNA (blue line) and protein-coding genes (red line).

Undoubtedly, high-throughput technologies enable the tremendous possibility to get both qualitative and quantitative information on whole transcripts mass produced by cells. This has resulted in high-resolution views of RNA expression dynamics throughout different tissues and time points [182–184] and fueled the development of ncRNA specific databases, such as Rfam [132], NRED [20], lncRNAdb [33], RNAdb [185], fRNAdb [186] and NONCODE [187]. Furthermore, various groups and projects, such as RefSeq [188], GENCODE [14,189], HAVANA team [190,191], Ensembl [192] and FANTOM [193] undertook the task to comprehensively annotate functional elements, including ncRNAs, of a number of species using experimental data. The RefSeq repository houses annotations resulting from automated analyses, collaboration and manual curation [188,194]. The GENCODE pipeline combines HAVANA and Ensembl automatic annotations to annotate the human gene features generated in the context of the ENCODE project [14,45,189]. The HAVANA team has the goal to provide manually curated annotations of transcripts aligned to human, mouse and zebrafish genomes. Ensembl runs an automatic *genebuild* process including *ab initio* gene predictions and release 64 supported a total of 61 species [192]. The Ensembl *genebuild* system is adapted to every species in the set according to the data that is available. For instance Ensembl imports and merges high quality HAVANA annotations exclusively for human and mouse. The FANTOM consortium aims to provide functional annotations to the full-length cDNAs [193]. The annotations generated by these consortia are freely available through genome browsers, including UCSC [195], Ensembl [196] and VEGA [197]. As new genomic regions get annotated and new transcript sequences become publicly available, these gene sets continue to growth [14,188,194]. A recent publication [14] indicated that in the last years the number of annotated protein-coding and non-coding transcripts in GENCODE has dramatically increased. For instance, passing from GENCODE version 3c (July 2009, http://www.gencodegenes.org/archive_stats.html) to version 7 (December 2010, http://www.gencodegenes.org/archive_stats.html), the number of protein-coding transcripts increased from 68,880 to 76,052, and the number of lncRNAs jumped from 10,457 to 15,512. In terms of gene annotations, the number of known protein-coding genes has remained almost unchanged, while the ncRNA gene annotations expanded tremendously (see Figure 4).

The overall picture, however, remains blurred by inconsistent findings, suggesting that more analyses are still needed. For instance, the recent estimates reported by the ENCODE project indicate that about the 62% of human genomic bases are expressed in long transcripts, while 5.5% only of the whole genome is found within the GENCODE annotated exons [198]. This discrepancy can be in part explained by the fact that GENCODE catalogues transcripts using cDNA/EST alignments [14] rather than RNA-seq short-read data. A classic low-throughput EST sequencing operated by the Sanger technology can identify mostly high abundant transcripts [199], while deep coverage RNA-seq experiments can reveal rare but potentially regulatory transcripts. Nonetheless, ESTs are longer than RNA-seq reads, and can provide more reliable transcriptional evidence [200].

### 4.3. NGS Challenges

To make the most out of the extraordinary possibilities that NGS offers, it is essential to understand the current limitations. One important point is that the reads returned by standard NGS platforms are usually short (35–500 base pairs [201]) and as a consequence it becomes necessary to reassemble the full-length transcripts. Small non-coding RNAs (*i.e.*, miRNA and piRNAs) represent an exception and there is no need to reassemble them, as they are small enough to be entirely covered by the read length. Unfortunately the process of reassembling transcriptomes starting from short reads is difficult. Normally RNA-seq dataset are big (gigabases to terabases), and thus need to be handled by sufficient large memory and by multi-CPU computers able to execute the algorithms in parallel with sufficient high-performance storage to store primary, temporary and output data. Although various short-read assemblers [202–204] were successfully applied to genome assembly, these packages cannot be easily used to reconstruct transcriptomes. Applying tools normally designed for genome reconstruction to the problem of transcriptome assembly leads to multiple complications. A key issue is that the DNA sequencing depth is supposed to be identical over the entire genome while transcriptome sequencing depth is expected to fluctuate significantly. For this reason, DNA short-read assemblers could erroneously interpret highly abundant transcripts as repetitive genomic regions. Furthermore, when using genome short-read assemblers the read strand is not taken into account. On the contrary, when available, a transcriptome assembler should exploit the strand information to unravel possible antisense expressions on different strands. Finally, the transcript modeling is involved as transcript variants coming from the same gene can share exons and are difficult to resolve unambiguously [199].

It is possible to work out the transcriptome assembly following a reference-based approach, a *de novo* assembly or combinations of each [199]. The first considers the initial mapping of the reads on a reference genome, and then the usage of transcript assemblers. To the end of labeling each read with the genomic location they come from, a new class of software, generally referred as read mapper, has recently shown up. In this context, the availability and the quality of the underlying reference genome are critical. Besides that, when dealing with massive amount of short-read data the CPU and the memory costs can be challenging, and several algorithms are being tailored to achieve best mapping efficiency [205–211]. Other important issues relate to the mapping of reads crossing exon-junction boundaries [212,213] and the uncertainty or lack of accuracy in read alignments. For most downstream applications, the accurate positioning of the reads back to the source genome is crucial. To improve the mapping accuracy, the process can take into account the read quality information [214,215]. The quality scores, introduced by the Phred algorithm [216,217], indicate the reliability of each base call in each read in a log-likelihood scale. Since bases with reduced quality scores have an increased probability to be sequencing errors, a read mapper should either use less severe penalization for mismatches at positions with low base-call quality, or not align such positions at all. The information about the quality score is particularly relevant when mapping reads of large size. This is a result of the fact that 3′ ends of longer reads are affected by sequencing errors at higher rates [215]. Besides choosing a threshold for accepted mismatches, other important and sometimes arbitrary decisions regard the split mapping and multiple mapping reads. The first refers to reads that could not be aligned to the reference genome unless split in subparts. Such reads could either highlight the presence of an unreported exon-junction boundary, or be sequencing artefacts. The second indicates reads that align multiple times across the reference genome. This mapping uncertainty is caused by repeated elements and may results in flawed expression establishments. On one hand, removing multiple mapping reads from the analysis would imply an underestimation of the expression of genes embedding repeats. On the other hand, considering multiple mapping reads would lead to artefactual expression measurements. Once mapped the reads, additional issues concern the application of transcript assemblers. Several computational tools have been developed with the purpose of reconstructing transcripts in their entire length, *i.e.*, annotating exon-intron transcript structures. These methods include Cufflinks [218], Isolazo [219] and Scripture [42]. In [220] the authors have shown that variations across transcript assemblers can be source of confusion, with low consistency across methods and a high number of false positives [200,219]. Transcript assemblers seem to have a better agreement when reconstructing protein-coding transcripts [43] with the agreement dropping dramatically when modeling large intervening ncRNAs (lincRNAs). For instance, Cufflinks and Scripture share only 46% agreement for lincRNA transcript models [43]. Such discrepancies are caused by the differences in how each assembler reconstruct lowly expressed transcripts [43]. In other words, about half of the isoforms estimated by a method in areas with low read density do not correspond to isoforms called by the other method. This poor agreement between transcript assemblers highlights the need for further improvements, calling for the development of new algorithms to accurately represent low abundant transcripts.

Another possibility to assemble a transcriptome from-short reads is *de novo* assembly of transcripts. This strategy does not require any reference genome and is therefore independent on the correct alignment of the reads to the splice sites. Advantages of this approach are that it is less reliant on accurate genome annotation and can be applied to organisms without sequenced or fully annotated genomes. Examples of applications adopting this strategy are described in [221–223]. Nevertheless, the application of *de novo* assembly to complex transcriptomes (e.g., higher eukaryotes) is complicated by the dataset sizes and the dense network of alternatively spliced variants. Furthermore, *de novo* transcriptome assemblers need much deeper sequencing than reference-based assemblers and are largely affected by sequencing errors [199].

Once transcriptome dataset is generated, there are additional complications in the downstream analysis if trying to distinguish genuine ncRNAs from mRNAs. Currently, this issue is becoming increasingly important as many researchers are only interested in one or the other. The most straightforward procedure would be to compare a newly generated transcriptome against existing gene annotations. However in most cases annotations are far from complete and the great majority of genes they include are protein-coding. As a consequence, in a normal RNA-seq experiment a substantial fraction of read contigs map outside of annotated exons [198]. Previously unreported transcripts can be either classified as ncRNA or mRNA according to the protein-coding potential they have. The *in-silico* assignment of a transcript to one of these two groups is not always trivial and may require dedicated expert curation [190]. Some transcript isoforms might insert coding exons and therefore could be partially translated, *i.e.*, generating small peptides. There are further ambiguities for coding transcripts whose untranslated structured molecules are also functional as ncRNAs [224] and for genes having both coding and non-coding isoforms [225]. A commonly used approach to predict the coding potential involves the codon substitution frequency (CSF) estimation [226,227]. This measure is based on an input multiple alignment of orthologous sequences. The CSF score deems a region to be coding depending on how the sequences of the multiple alignment evolved, *i.e.*, showing distinctive mutation patterns, as are expected in coding and non-coding loci. A coding region is expected to embed prevalently conservative amino acid substitutions and synonymous codon substitutions, while showing low occurrence of nonsense and missense mutations. Although CSF has been successfully applied in various research projects [226,228,229], the score is not always easy to estimate with the availability of trustworthy orthologues being the main limiting factor when dealing with new transcriptome datasets. Issues include scarcity or even the absence of orthologs, erroneous insert of pseudogenes in the set and absence of informative variations. For instance, as shown in [40] many putative human lncRNAs are not found in other species, and cannot be analyzed using CSF. Besides this, primate specific lncRNAs rarely show sufficient changes to highlight sense/nonsense mutations patterns. In addition to CSF, other strategies not relying on evolutionary signatures can be effectively used to predict if a transcript is going to be translated into protein or not. For example, there are dedicated BLAST flavors including BLASTx and RPS-Blast [124,230] that can be used to identify transcripts whose translational product possesses a match in protein databases such as Pfam [133] and UniProt [231]. Other algorithms include CPC (Coding Potential Calculator), a support vector machine (SVM) classifier including both Open Reading Frame (ORF) and homology predictions features [232], PORTRAIT (Prediction of transcriptomic ncRNA by ab initio methods) a SVM classifier not using homology information [233] and CPAT (Coding Potential Assessment Tool), a logistic regression model built with four sequence features including ORF predictions [234]. Unfortunately, bioinformatics predictions can easily return mistaken assignments when dealing with ncRNAs closely related to coding mRNAs, and result in some confusion when transferring annotation across species, or within a genome. Such observations may wrongly suggest pseudogenization events or a turnover between proteins and ncRNAs.

### 4.4. Other Approaches

Over the last few years, other approaches alternative or complementary to RNA-seq have been attempted to generate high-throughput ncRNA annotations. In 2009, Mitchell Guttman and co-workers published the first of a series of analysis that recently came out linking lncRNA detection to histone modifications [13]. In this work, the authors pioneered a chromatin-state based method to identify well-defined transcriptional units occurring between known protein-coding genes. Their analysis relied on the observation by [235] that promoters of genes expressed by the RNA polymerase II (Pol II) are signed by trimethylation of lysine 4 of histone H3 (H3K4me3) while the transcribed area is marked by trimethylation of lysine 36 of histone H3 (H3K36me3). Following this observation, the authors did chromatin immunoprecipitation followed by high-throughput sequencing (ChIP-seq) [235] to generate profiles of chromatin states. This approach revealed 1600 mouse lincRNAs, corresponding to H3K4me3-H3K36me3 chromatin domains and lying outside of protein-coding regions. The prediction reliability has been estimated by additional analysis showing that lincRNAs are more conserved than neutrally evolving sequences and that most of experimentally tested loci were found to be expressed [13]. An alternative strategy used for ncRNA detection involves a combination of different high-throughput data sources and their integration using bioinformatics [236]. This approach, named incRNA, relies on a machine learning method and has been applied to the genome-wide identification of *Caenorhabditis elegans* ncRNAs. incRNA combines predicted and experimental data for a total of nine different information sources. These include the expression data coming from various developmental stages and conditions, as well as the GC content, the predictions of RNA secondary structure folding energy, the prediction of evolutionary conserved DNA sequence and secondary structure. These results illustrate how the integration of multiple information sources ends in highly accurate predictions of novel ncRNA genes.

Recently, a number of works reporting a massive quantity of novel ncRNA genes in various species have been published [40,43,44,237,238]. Such rapid growth has been possible thanks to the parallel development of new and ever more sophisticated bioinformatics approaches. Nevertheless, such analyses remain superficial with uncertainties of different type and degree affecting most predictions. For example, the homology search pipeline described in [40] is not sensitive enough to map rapidly evolving lncRNAs, hence the limit to play comprehensive evolutionary study. Moreover such lncRNA predictions should be taken with care, not just because they are not experimentally verified, but also because they are far from representing the complete genome-wide lncRNA figure. For validation purpose, some works provide the number of predicted lncRNA supported by expression evidences. For instance in [13] the authors confirmed by tiling array the expression of ~70% lncRNA predictions. In other cases as in [44], RT-PCR has been used to validate 15 newly identified lncRNAs. On the short run available transcription data is expected to increase very rapidly, and the necessity to accurately and quickly validate ncRNAs is becoming more pressing than ever.

## 5. Discussion and Conclusions

ncRNA functional characterization is a rapidly expanding research area. In the past few years, it has become clear that the majority of the transcripts in cells are more than mere intermediates between the hereditary information encoded in DNA and the mechanical operative component represented by proteins. Indeed, it appears that numerous transcripts may not be translated at all while still being involved in critical biological functions such as cell differentiation and chromatin remodeling. Taking together 15 human cell lines, the cumulative coverage of transcribed regions is ~62% and ~75% of the whole human genome for processed and primary transcripts, respectively [239]. This “pervasive transcription” is strikingly high, especially when considering that a mere 3% of the human genome codes for protein-coding exons. [198]. Numerous novel, previously uncharacterized RNA species have been recently detected. A sizeable fraction of them are defined as lncRNA, *i.e.*, functional molecules longer than 200 nucleotides that do not show any coding potential. Some of these molecules are spliced, capped, differentially expressed in tissues/cells or developmental stages and tend to be more conserved across species than would result from neutral evolution. For these reasons and because of the increasing number of transcripts whose function has been experimentally validated, it is believed that many of these new ncRNAs belong to an important, relatively unexplored class of regulatory elements. Thanks to ongoing improvements in sequencing technologies it has become possible to collect a significant amount of these uncharacterized transcripts. The latest generation of sequencing technologies makes it possible to perform large scale sequencing of entire transcriptomes. This technique, known as RNA-seq has already had a dramatic impact on our perception of the human transcription landscape [183,239]. Similar studies have been carried out in a number of genetic model organisms including rodents [44,151], plants [240], insects [184], worms [241] and yeasts [242]. In [243] the author argues that RNA-seq represents the most promising technology for transcriptome research. The main strength of RNA-seq approaches are the high degree of dynamic range they offers, returning better sensitivities than microarrays without the need of *a priori* speculation regarding the genomic loci being transcribed [244]. If the pace of scientific progress is maintained and if costs keep decreasing, one can reasonably expect this technology to rapidly become a key component of personalized medicine, especially when considering the new venues of development that are currently being considered [152,245].

From a functional perspective, much remains to be done for accurate characterization and functional analysis of ncRNAs. To infer the function of novel ncRNAs one possibility is looking for functional motifs. This can be done by running motif finders algorithms to predict structurally conserved and potentially functional motifs [246–252]. Furthermore, the functional characterization of a novel ncRNA can be aided by the detection of protein-RNA binding motifs and the identification of protein interaction partners. Experimental approaches suited for this include RIP (Rna Immunoprecipitation) and CLIP (Cross-Linking and ImmunoPrecipitation) [253]. Comparative studies also offer a very efficient way to have functional insights and prioritizing analysis. They can be used to predict function by homology, assess phylogenetic relationships, detect functional motifs or classify related molecules in order to identify families. A major challenge when tackling ncRNA comparisons results from the remarkable variability of traits and functions. Considering sizes only, ncRNA molecules can be as short as a miRNA (~22 nt) and up to ~17 kb long in the case of Xist [2]. Another difficulty when comparing ncRNAs is that most of these genes have poorly conserved sequences. Such diversity challenges our ability to compare, classify and search with conventional alignment tools. In addition ncRNA genes have no equivalent of codon bias and ORFs that help powering the statistical component of machine learning approaches when doing protein prediction [254]. The strongest signal contained by RNA sequences is usually evolutionarily conserved secondary structures. Many efficient algorithms exist that are able to predict potential structures using MFE or SCFG computations. Unfortunately, these predictions ignore the contribution of the environment and are not always accurate enough to significantly improve alignment accuracy and homology modeling. Emerging technologies allowing the high-throughput generation of experimentally derived secondary structures [255] will hopefully help addressing this problem. Unfortunately, taking into account secondary structures while comparing sequences is a challenging procedure, too intensive from a computational point of view to be practical in most circumstances [108]. This makes it is difficult to compare mono-exonic genes while taking the secondary structure into account, and totally impossible when the transcripts are multi-exonic (*i.e.,* the secondary structures are interrupted by introns). It has been shown [40,237] that BLAST can be effectively used for lncRNA homolog prediction, in combination with splicing informed heuristics such as exonerate [256] or GeneWise [257]. This strategy is not new, and similar approaches have already been used for the discovery of protein-coding homologs [258–260]. As one would expect, homology based RNA searches are severely limited by the capacity to align distant homologues. For instance, when searching the human lncRNA complement against mammalian genomes [40] or when using an estimated pig complement [237], the authors only managed to find, beyond primates, less than 50% of the query genes across cow, mouse or dog. This result may reflect a high turnover, but the conservation/disappearance patterns, poorly correlated to phylogenetic history, are most likely indicative of a limited detection capacity. Other confounding factors include misassembled or partially sequenced genomes. Additional analysis would be needed to validate the Blast/exonerate mapping approach. At this stage, it is therefore impossible, without further experiments, to establish whether lncRNA queries that failed to map are really absent in the target species or undetected. In this context, high quality templates, such as the GENCODE queries used in [40], offer better likelihood to return precise annotation. In the same publication it is also predicted that sizeable fraction of the human lncRNAs is primate specific [40]. This result is in agreement with a recently published study [44] where the authors identified lncRNAs expressed in rodents’ adult liver, and then compared the expression of the orthologous genomic regions. This work illustrates that loss of lncRNA transcription among rodents is associated with loss of sequence constraints and that many lncRNA genes seems to be species or lineage specific. Another application of homology based approaches is the possibility to identify novel human lncRNA genes candidates by using non-human templates as query [237]. As shown in the paper [237], there are 131 pig lncRNAs mapping to unannotated regions of the human genome. This result suggests that although human is probably one of the most extensively annotated higher-eukaryote, extra improvements might be achieved using data gathered in other non-model organisms. In [40] the authors also extend the lncRNA conservation study to a multiple genome alignments strategy based on PhastCons conservation scores. The analysis is in agreement with previous reports [13,30] and confirms that lncRNAs sequences are less constrained than those of protein-coding genes. Remarkably, it was shown that the distribution of lncRNA exons conservation is bimodal, with a fraction substantially approximate to ancestral repeats, and another group appreciably shifted toward the protein-coding set. This indicates that some lncRNA are under a selection as strong as that seen for proteins and suggests that a sizeable fraction of lncRNA genes are probably functional. The fraction of lncRNAs having a mutation rate almost indistinguishable from repeats suggests that at least some lncRNAs (close to a third) may be transcriptional noise. However, despite this abundance of lncRNA sequences that do not appear to be under selection, the transcript product itself might still have a biological role and as shown in [261,262] the transcription process itself of some ncRNA can bear regulative functions.

Despite the difficulties encountered when comparing ncRNAs, homology search of ncRNAs can be successfully used to detect new genes. New and ever more sophisticated algorithms will help addressing the challenges brought by NGS technologies. The ultimate goal is the creation of thorough transcriptome annotations and unbiased expression profiling of each individual transcript. It is still too early to tell. However, if they live up to their promises and expectation, the discovery of this new large class of RNAs may well define one of the turning points of modern biology.

## Figures and Tables

**Table 1:** A summary of methods, datasets and browsers for non-coding RNA analysis. The first column indicates the resource type. The second column the resource name. The third column reports the PubMed ID when available, if not the web address. The fourth column provides a brief description of the resource.

	Resource	Pubmed ID	Description
Comparing ncRNAs (Section 2)	Mfold	6163133	Single sequence RNA secondary structure prediction.
	RNAfold	12824340	
	WAR	18492721	WEB server allowing the execution of different alignment methods
	RNAalifold	12079347	Folding previously aligned RNAs (Plan A)
	PFOLD	12824339	
	ILM	14693809	
	Construct	10518612	
	Dynalign	11902836	Sankoff derived algorithm for the simultaneous alignment and secondary structure prediction (Plan B)
	Foldalign	9278497	
	Stemloc	15790387	
	Consan	16952317	
	pmmulti	15073017	
	R-Coffee	18420654	Aligners taking into account previously estimated secondary structure (Plan C)
	RNAcast	16020472	
	SARA	18689811	3D structure alignment method
	DIAL	17567620	
	iPARTS	20507908	
	ARTS	16204124	
	SARSA	18502774	
	LaJolla	http://www.mdpi.com/1999-4893/2/2/692	
	FRASS	20553602	
Detecting ncRNAs (section 3)	ML-heuristic	16267089	Profile HMM
	RAGA	9358168	Genetic algorithm
	RSEARCH	14499004	Covariance model
	Infernal	12095421	
	BlastR	21624887	BLAST-based dinucleotide homology search
Datasets and browsers (section 4)	ENCODE	22955616	Consortium
	Ensembl	22086963	
	FANTOM	11217851	
	HAVANA	http://www.sanger.ac.uk/research/projects/vertebrategenome/havana/	Annotation team
	GENCODE	22955987	Project for the annotation of all human gene features
	UCSC	12045153	Genome browser
	VEGA	18003653	
	RefSeq	18927115	Collection of DNA, transcripts, and proteins
	Rfam	12520045	ncRNA database
	NRED	18829717	
	lncRNAdb	21112873	
	RNAdb	17145715	
	fRNAdb	17099231	
	NONCODE	15608158	
